# Point/counterpoint: We should not take the direction of blood pressure change into consideration for dynamic cerebral autoregulation quantification

**DOI:** 10.1177/0271678X221123442

**Published:** 2022-09-13

**Authors:** Kyriaki Kostoglou, David M Simpson, Stephen J Payne

**Affiliations:** 1 Institute of Neural Engineering, Graz University of Technology, Graz, Austria; 2 ISVR, Faculty of Engineering and the Environment, University of Southampton, Southampton, UK; 3 Institute of Applied Mechanics, National Taiwan University, Taipei

**Keywords:** Asymmetry, cerebral autoregulation, cerebral blood flow, cerebral haemodynamics, transcranial Doppler

## Abstract

Over the past years, a wide range of studies have provided evidence of asymmetry in the response of static and dynamic cerebral autoregulation (CA) during increasing and decreasing pressure challenges. The main message is that CA is stronger during transient increases of arterial blood pressure rather than decreases. Here we do not argue against the presence of CA asymmetry but we seek to raise questions regarding the measurement of the effect and whether this effect needs to be taken into account, especially in clinical settings.

Autoregulation of cerebral blood flow (CBF), also known as cerebral autoregulation (CA), denotes the intrinsic ability of the brain to protect itself against changes in arterial blood pressure (BP). Lately, one research focus has shifted towards the asymmetric behavior of CA during BP increases and decreases. Specifically, evidence suggests that both static and dynamic CA (dCA) respond differently during increasing and decreasing pressure challenges.^1–9^ For example, Aaslid et al.^1^ observed a strongly asymmetric dCA response in patients with traumatic brain injury. Tzeng et al.^2^ reported dCA asymmetry in healthy participants during pharmacologically induced BP fluctuations. Brassard et al.^3^ and Panerai et al.^4^ also support the existence of dCA asymmetry in healthy male volunteers. It is worth mentioning that there is also evidence of asymmetry from studies of the related baroreceptor mechanism for BP control.^6^ Given the complexity of CA mechanisms, it would be difficult to expect CA to be perfectly symmetrical in its responses: symmetry is a mathematical convenience but not necessarily of benefit in physiological systems.

Asymmetry has been mainly observed during large BP fluctuations induced by controlled conditions (e.g. thigh-cuff deflation, squat-stand maneuvers, pharmacological intervention).^1–5^ Spontaneous BP oscillations,^7^ though, have shown to produce weaker asymmetric responses. In preliminary work, Simpson et al.^8^ did observe poor agreement between dCA indices from increasing and decreasing BP sequences. However, the interpretation of results reinforces the observation that asymmetry is difficult to accurately quantify using small fluctuations. Since spontaneous oscillations are the preferred (and sometimes only feasible) protocol in patients, it may be difficult to measure, let alone to exploit, asymmetry in the clinical setting.

To assess asymmetry, the data is usually separated into segments of increasing or decreasing BP, respectively. CA indices (based on the BP and CBF velocity (CBV) relationship) are then extracted from each and compared. The reduced signal length in each segment increases random estimation errors in CA parameters which needs to be balanced against any increase in accuracy from removing the assumption of symmetry – or compensated by extending recording times. Biases may also arise due to the choice of the experimental protocol. For example, during squat-stand maneuvers,^5^ the BP increases are much faster than the decreases, and there is a risk of confounding between the effects of direction and dynamics (e.g. rate of change) of BP in assessing CA. Recent studies have tried to compensate for differing dynamics,^9^ nonetheless the crucial question of defining the meaning of CA asymmetry remains open. Perhaps the most obvious interpretation is that with a symmetrical response, if the BP signal were flipped upside down, the CBV signal would simply be flipped upside down too. This would be observed if CA behaved as a linear filter model (as assumed in transfer function analysis, finite/infinite impulse response models or autoregulation index [ARI]) but also with some nonlinear models (e.g. odd-numbered polynomials). If BP changes are asymmetrical, as probably most real-world challenges are, then asymmetry in the resultant CBV is to be expected. However, we would hesitate to refer to this as evidence for asymmetry in the response, given the asymmetry in the challenge. Furthermore, CA responses to BP changes cannot be independent: if the attenuation of CBV response to BP increases is stronger than that for decreases, then repeated squat-stand maneuvers would lead to a gradual decrease in CBV (due to a smaller increase in CBV than the subsequent decrease, in successive cycles) – which is not evident in the currently available data; asymmetry could however still be present in the speed of the response (e.g. faster CBV responses to increasing than decreasing BP). Other physiological variables, such as arterial pCO2, that may also be impacted by the maneuvers, could further confound the analysis. The focus on BP increase and decrease may also be a simplification that neglects other aspects of the complex physiology, such as the BP and CBV baseline values, especially during experimental maneuvers. Thus, greater clarity in the definition of asymmetry is still required, to develop this debate further.

A more complete analysis of the asymmetry probably necessitates the use of more sophisticated mathematical models^10^ than those that are currently used to assess autoregulatory efficacy (e.g. nonlinear/time-varying models). Following such analysis, the question however remains as to what type of indices/biomarkers can be extracted from these models to accurately quantify asymmetry. It is not clear that we currently understand the clinical importance of CA sufficiently to do this in the most relevant manner, for example when considering raising or lowering a patient’s blood pressure.

We have argued that while asymmetry has been repeatedly observed, the results may be influenced by measurement methods, which might lead to some confounding, such as from differing speeds of BP changes, changes in baseline BP and CBV and fluctuations in other relevant physiological variables. The question remains whether the autoregulatory response depends primarily on the direction of BP changes or other factors – the definition of asymmetry needs to be refined. Although CA asymmetry is clearly of physiological interest, we still lack evidence of its diagnostic and prognostic value. Further work with more tightly controlled experimental conditions is needed to assess whether any observed asymmetry reflects specific physiological or pathological conditions and whether this adds value when investigating different (especially clinical) populations and hence if and when (and how) it should be routinely incorporated into CA analysis.
